# ACADEMIC PARTNERING IN A COMMUNITY-BASED FALL PREVENTION PROGRAM

**DOI:** 10.1093/geroni/igz038.1767

**Published:** 2019-11-08

**Authors:** Dennis W Klima, Emily Wehlandl, Aspen Holmes, Jessica Weimert, Nicholas Rhoten, Jestine Wolfe, Katherine Avila, Nate Austin

**Affiliations:** UMES, Princess Anne, Maryland, United States

## Abstract

Falls are a major cause of disability nationally are and linked to both fractures and fear of falling. The purpose of this study was to assess the effectiveness of a rural community-based fall prevention program using an academic partnership model with seven physical therapy students rotating through six senior centers. One hundred and fifty-four older adults (Mean age: 76.1+/- 8.5) completed Stepping On at six locations. A mixed-methods design was utilized. Physical therapy students partnered with older adult participants to teach exercises, strategize floor recovery techniques, and identify community safety barriers. A descriptive survey tool assessed demographic profiles, falls efficacy, and program effectiveness among participants. Students participated in a follow-up focus group to discuss perspectives on their role in the fall prevention program. Most participants were female (86.4%), lived alone (50.0%) and taking four or more medications (74%). Thirty-eight participants (24.7%) had fallen over the past year. Eighty-eight (57.1%) subjects noted they had less fear of falling following the community-based intervention; moreover, most subjects (74.7%) reported having an improved plan for floor rise after a fall. Major focus group themes underscored students’ enhanced ability to teach exercise and mobility activities with an increased awareness of interdisciplinary fall prevention. Following a collaborative community-based fall prevention program, seniors have a better understanding of fall causes and plan to seek floor recovery assistance. In turn, student teaching and communication skills are reinforced. Student partnering with seniors promotes fall prevention strategies and affords benefits to both students and participants.

